# Optimal treatment strategy for hormone receptor-positive human epidermal growth factor receptor 2-negative breast cancer patients with 1–2 suspicious axillary lymph node metastases on breast magnetic resonance imaging: upfront surgery vs. neoadjuvant chemotherapy

**DOI:** 10.3389/fonc.2023.936148

**Published:** 2023-05-17

**Authors:** Seung Eun Lee, Sung Gwe Ahn, Jung Hwan Ji, Yoonwon Kook, Ji Soo Jang, Seung Ho Baek, Joon Jeong, Soong June Bae

**Affiliations:** ^1^ Department of Surgery, Gangnam Severance Hospital, Yonsei University College of Medicine, Seoul, Republic of Korea; ^2^ Institute for Breast Cancer Precision Medicine, Yonsei University College of Medicine, Seoul, Republic of Korea; ^3^ Department of Surgery, Catholic Kwandong University International St. Mary’s Hospital, Incheon, Republic of Korea

**Keywords:** breast neoplasm, breast MRI, axillary lymph node metastasis, upfront surgery, neoadjuvant chemotherapy

## Abstract

**Background:**

It is unclear whether upfront surgery or neoadjuvant chemotherapy is appropriate for first treatment in hormone receptor (HR)-positive human epidermal growth factor receptor 2 (HER2)-negative breast cancer patients with 1–2 suspicious axillary lymph node (ALN) metastases on preoperative breast magnetic resonance imaging (MRI).

**Method:**

We identified 282 patients with HR+HER2- breast cancer and 1–2 suspicious ALN metastases on baseline breast MRI (147 received upfront surgery; 135 received neoadjuvant chemotherapy). We evaluated the predictive clinicopathological factors for pN2-3 in the adjuvant setting and axillary pathologic complete response (pCR) in the neoadjuvant setting.

**Results:**

Lymphovascular invasion (LVI)-positive and clinical tumors >3 cm were significantly associated with pN2-3 in patients who received upfront surgery. The pN2-3 rate was 9.3% in patients with a clinical tumor ≤ 3 cm and LVI-negative versus 34.7% in the others (p < 0.001). The pN2-3 rate in patients with a clinical tumor ≤ 3 cm and LVI-negative and in the others were 9.3% versus 34.7% in all patients (p < 0.001), 10.7% versus 40.0% (p = 0.033) in patients aged < 50 years, and 8.5% versus 31.0% in patients aged ≥ 50 years (p < 0.001), respectively. In the neoadjuvant setting, patients with tumor-infiltrating lymphocytes (TILs) ≥ 20% had a higher axillary pCR than those with TILs < 20% (46.7% vs. 15.3%, p < 0.001). A similar significant finding was also observed in patients < 50 years.

**Conclusions:**

Upfront surgery may be preferable for patients aged ≥ 50 years with a clinical tumor < 3 cm and LVI-negative, while neoadjuvant chemotherapy may be preferable for those aged < 50 years with TILs ≥ 20%.

## Background

Neoadjuvant chemotherapy is widely adopted as the standard of care for primary operable breast cancer (1). An excellent treatment response, such as pathologic complete response (pCR) or low residual cancer burden, is well known as associated with a favorable prognosis (2–4). Furthermore, the response to neoadjuvant chemotherapy provides helpful information about adjuvant treatment (5, 6). In addition, neoadjuvant chemotherapy can allow breast cancer downstaging and has led to an increase in breast-conserving surgery and a decrease in unnecessary axillary lymph node dissection (ALND) (7). Previous studies suggested that ALND can be omitted if no metastasis to three or more sentinel lymph nodes is noted after neoadjuvant chemotherapy (8–10). However, the response to neoadjuvant chemotherapy is poor in hormone receptor (HR)-positive human epidermal growth factor receptor 2 (HER2)-negative breast cancer compared to HER2+ breast cancer or triple-negative breast cancer (TNBC) (3).

Although chemotherapy has contributed to a decline in breast cancer mortality, it is associated with severe toxicities that reduce the quality of life. Because HR+HER2- breast cancer has a favorable prognosis, multigene assays have been increasingly applied to affected patients without metastasis of axillary lymph nodes (ALNs) to avoid overtreatment (11, 12). Furthermore, chemotherapy can be omitted in postmenopausal women with 1–3 positive ALNs who have low Oncotype DX recurrence score based on the RxPONDER trial results (13).

Therefore, neoadjuvant chemotherapy may be preferable for patients with HR+HER2- breast cancer with ≥4 metastatic ALNs. Furthermore, in such cases, neoadjuvant chemotherapy provides an opportunity to avoid ALND according to axillary treatment response. Conversely, upfront surgery may be preferable in patients with HR+HER2- breast cancer with N0-1 owing to the possibility of omitting chemotherapy through multigene assays, especially in postmenopausal women. However, in clinical practice, it is not easy for physicians to choose the optimal treatment sequence (upfront surgery vs. neoadjuvant chemotherapy) for HR+HER2- breast cancer with 1–2 suspicious ALNs on radiologic modalities because the actual nodal burden is unknown.

To determine the proper treatment strategy for patients with HR+HER2- breast cancer who had 1–2 suspicious ALNs on pretreatment breast magnetic resonance imaging (MRI), we explored the clinicopathologic factors related to multiple ALNs metastases in patients who underwent upfront surgery and axillary pCR in patients who received neoadjuvant chemotherapy.

## Methods

### Study population

The study protocol was reviewed and approved by the Institutional Review Board (IRB) of Gangnam Severance Hospital, Yonsei University, Seoul, Korea and adhered to the tenets of the Declaration of Helsinki (IRB no. 3-2021-0155). The IRB waived the requirement for written informed consent owing to the study’s retrospective design.

**Figure 1:**
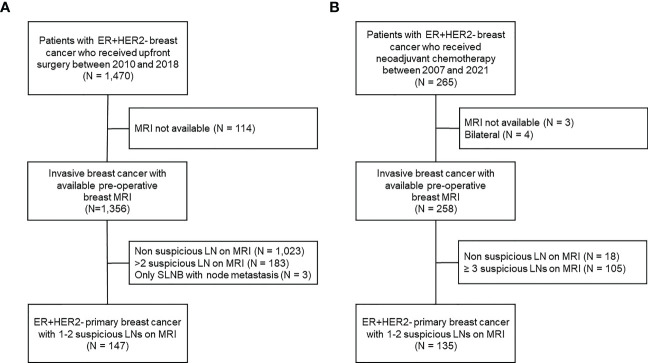
Flowchart of patient enrollment and exclusion criteria. **(A)** Patients who received upfront surgery, **(B)** patients who received neoadjuvant chemotherapy.

Between January 2007 and July 2021, we identified patients with HR+HER2- invasive breast cancer treated at Gangnam Severance Hospital. The exclusion criteria were as follows: bilateral breast cancer, unavailability of preoperative breast MRI, and no suspicious axillary lymph nodes or > 2 suspicious ALNs on preoperative breast MRI. Among the patients who underwent upfront surgery, all patients received sentinel lymph node biopsy (SLNB), and additional ALND was performed only when metastasis was confirmed in SLNB. To identify the patients whose pN stage was accurately evaluated, cases in which sentinel lymph node metastasis but only SLNB was performed were excluded. Finally, 147 patients who underwent upfront surgery and 135 who received neoadjuvant chemotherapy were included in this study (**Figure 1**).

Clinical data, including age at diagnosis, breast and axillary surgery, neoadjuvant chemotherapy, and response to treatment, were obtained from the electronic medical records. Pathologic data, such as histologic type, histologic grade, lymphovascular invasion (LVI), estrogen receptor (ER), progesterone receptor (PR), Ki-67, tumor-infiltrating lymphocytes (TILs), and nodal metastases, were obtained from surgical specimens in the adjuvant setting. Pathologic data, including ER, PR, and TILs, were evaluated using pre-treatment core biopsy samples in the neoadjuvant setting.

### Breast MRI

Breast MRI was performed in all patients using a 3.0-T MR scanner (Achieva; Philips Medical System, Best, Netherlands) with a dedicated sensitivity encoding-enabled, four-channel breast coil. Patients were examined in the prone position. All images were acquired from bilateral axial views. The turbo spin-echo T1-and T2-weighted sequences and T2-weighted fat-suppressed spin-echo series were included in the routine protocol. One pre-contrast and five post-contrast series were included in the dynamic contrast-enhanced MRI examination using a fat-suppressed T1-weighted gradient-echo sequence (repetition time/echo time, 4.9/2.4; matrix, 340 × 340; flip angle, 12°; field of view, 34 × 34 cm; slice thickness, 1.5 mm). Gadobutrol (Gadovist, Bayer Healthcare, Berlin, Germany), at a dose of 0.1 mmol/kg, was injected using an automated injector (Nemoto; Nemoto Kyorindo, Tokyo, Japan) at a rate of 2 mL/s, followed by a 20-mL saline flush. The acquisition time for each post-contrast series was 74 seconds (14).

MR images of ALN and index breast cancer obtained before treatment were reviewed. Suspicious ALN features were irregular margins, round shape, eccentric cortical thickening, and loss of fatty hilum. If one or more of the above-mentioned suspicious features was present, the ALNs were considered suspicious and their numbers counted. For index breast cancer, the clinical tumor size was determined by the largest dimension.

### Pathology

We defined LVI based on the Columbia University Irving Medical Center standard pathological definition of the presence of carcinoma cells within a definite endothelial-lined space, including lymphatic or blood vessels. This was verified using D2-40 immunohistochemical staining for the lymphatic endothelium and CD31 for the endothelium of all vessels. The presence of LVI was evaluated of the surgical pathology specimens.

Stromal TILs were evaluated using all cores containing invasive tumor cells from the biopsy samples or surgical specimens according to the TIL Working Group assessment guidelines (15). The mononuclear cells including lymphocytes and plasma cells, except for polymorphonuclear leukocytes, were counted and the average score was reported as a percentage (16). TIL levels were categorized as high (≥20%) or low (<20%).

In the adjuvant setting, pN categories were determined according to the anatomical stage of the American Join Committee on Cancer guidelines (8^th^ edition); i) pN0, no regional axillary lymph node metastasis identified or isolated tumor cell custers only, ii) pN1, micro- or macrometastases in 1-3 axillary lymph nodes, iii) pN2, metastases in 4-9 axillary lymph nodes, iv) pN3, metastases in 10 or more axillary lymph nodes. Axillary pCR (ypN0) was defined as the complete absence of invasive tumors, including micrometastasis, and isolated tumor cells in surgical specimens after neoadjuvant chemotherapy.

### Statistical analysis

The principal outcome was to identify the predictive factors for pN2-3 in the adjuvant setting and axillary pCR in the neoadjuvant setting. Baseline characteristics were compared according to multiple ALN metastases or axillary pCR using the chi-squared test or Fisher’s exact test for categorical variables. P-values < 0.05 were considered statistically significant. We also used binary logistic regression models to identify the significant clinicopathological factors related to multiple ALN metastases. Odds ratios (ORs) and 95% confidence intervals (CIs), with a two-sided p-value, are presented. All statistical analyses were performed using IBM SPSS Statistics (version 25.0; SPSS, Chicago, IL, USA).

## Results

### Nodal burden in patients who received upfront surgery

The baseline characteristics of the 147 patients who underwent upfront surgery are summarized in **Table 1**. Of these patients, 32 (21.8%) patients had pN2-3, and 115 (78.2%) had pN0-1. Among the 115 patients with pN0-1, 55 (47.8%) had no metastatic axillary lymph nodes, and 60 (52.2%) patients had pN1. Accordingly, the sensitivity of breast MRI for axillary lymph node metastasis was 62.5% in this cohort. In addition, 55 patients with pN0 underwent only SLNB, while 92 patients with pN1-3 underwent SLNB followed by ALND.

**Table 1:** Clinicopathologic characteristics of the patients who received upfront surgery according to axillary lymph node metastasis.

Variables	pN0-1 (n=115)	pN2-3 (n=32)	Total (n=147)	P-value
Age				0.332
<50	43 (37.4)	15 (46.9)	58 (39.5)	
≥50	72 (62.6)	17 (53.1)	89 (60.5)	
Multiple mass				0.120
no	69 (60.0)	24 (75.0)	93 (63.3)	
yes	46 (40.0)	8 (25.0)	54 (36.7)	
Breast surgery				0.032
BCS	53 (46.1)	8 (25.0)	61 (41.5)	
Mastectomy	62 (53.9)	24 (75.0)	86 (58.5)	
Axillary surgery				<0.001
SLNB	55 (47.8)	0	55 (37.4)	
ALND	60 (52.2)	32	92 (62.6)	
Histologic type				0.332
IDC	92 (80.0)	28 (87.5)	120 (81.6)	
Others	23 (20.0)	4 (12.5)	27 (18.4)	
HG				0.149
1	24 (20.9)	5 (15.6)	29 (19.7)	
2	75 (65.2)	26 (81.3)	101 (68.7)	
3	16 (13.9)	1 (3.1)	17 (11.6)	
LVI				<0.001
no	83 (72.2)	11 (34.4)	94 (63.9)	
yes	32 (27.8)	21 (65.6)	53 (36.1)	
PR				0.409^*^
positive	96 (83.5)	29 (90.6)	125 (85.0)	
negative	19 (16.5)	3 (9.4)	22 (15.0)	
Ki-67				0.776
<14	75 (65.2)	20 (62.5)	95 (64.6)	
≥14	40 (34.8)	12 (37.5)	52 (35.4)	
TILs^†^				0.210
<20%	27 (64.3)	10 (83.3)	37 (68.5)	
≥20%	15 (35.7)	2 (16.7)	17 (31.5)	
Clinical tumor size				0.031
≤3cm	90 (78.3)	19 (59.4)	105 (71.4)	
>3cm	25 (21.7)	13 (40.6)	42 (28.6)	
No of metastatic LN				<0.001
0	55 (47.8)	0	55 (37.4)	
pN1	60 (52.2)	0	60 (40.8)	
pN2	0	32 (100.0)	32 (21.8)	

Unless otherwise noted, values are the number of patients, with percentages in parentheses.

^*^P-value was obtained with the Fisher’s exact test.

^†^Missing values.

BCS, breast-conserving surgery; SLNB, sentinel lymph node biopsy; ALND, axillary lymph node dissection; IDC, invasive ductal carcinoma; HG, histologic grade; LVI, lymphovascular invasion; PR, progesterone receptor; TILs, tumor-infiltrating lymphocytes; LN, lymph node.

The LVI and clinical tumor size (cT) were significantly associated with pN2-3; more LVI-positive (65.6% vs. 27.8%, p < 0.001) and cT > 3 cm (40.6% vs. 21.7%, p = 0.031) were found in the pN2-3 group compared to the pN0-1 group (**Table 1**). Furthermore, the multivariable analysis showed that LVI-positive status (OR, 4.68; 95% CIs, 1.96–11.16; p = 0.001) was an independent factor for pN2-3 (**Table 2**). The remaining variables did not differ between the two groups. Fine-needle aspiration biopsy (FNAB) was performed in 45 (30.6%) of 147 patients. The pN2-3 rate was 45.7% in FNAB-positive cases, while 5.9% in FNAB-negative cases (**Supplementary Table 1**).

**Table 2:** Univariable and multivariable analysis of predictors for pN2-3.

Variables	Univariable		Multivariable	
	OR (95% CIs)	P-value	OR (95% CIs)	P-value
Age				
<50	Ref.		Ref.	
≥50	0.68 (0.31-1.49)	0.333	0.68 (0.27-1.69)	0.405
Multiple mass				
no	Ref.		Ref.	
yes	0.38 (0.08-1.76)		0.33 (0.07-1.69)	0.184
Histologic type				
IDC	Ref.		Ref.	
Others	0.57 (0.18-1.79)	0.337	0.53 (0.15-1.91)	0.333
HG				
1 or 2	Ref.		Ref.	
3	0.90 (0.10-8.30)	0.922	1.06 (0.10-11.44)	0.959
LVI				
no	Ref.		Ref.	
yes	4.95 (2.15-11.42)	<0.001	4.68 (1.96-11.16)	0.001
PR				
positive	Ref.		Ref.	
negative	1.91 (0.53-6.93)	0.323	2.27 (0.50-10.36)	0.29
Ki-67				
<14	Ref.		Ref.	
≥14	1.13 (0.50-2.53)	0.776	1.10 (0.42-2.84)	0.841
Clinical tumor size				
≤3cm	Ref.		Ref.	
>3cm	2.46 (1.07-5.67)	0.034	1.95 (0.79-4.85)	0.149

OR, odds ratio; CIs, confidence intervals; IDC, invasive ductal carcinoma; HG, histologic grade; LVI, lymphovascular invasion; PR, progesterone receptor; Ref, reference.

**Figure 2:**
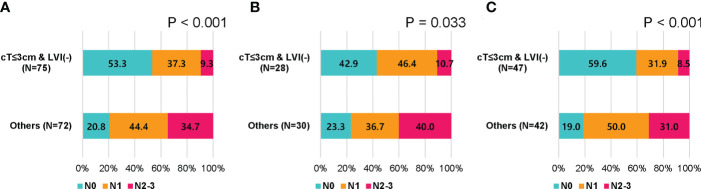
Nodal stage by clinical tumor size and lymphovascular invasion in patients who received upfront surgery. **(A)** All patients, **(B)** patients younger than 50 years, and **(C)** patients of 50 years or older.

Based on these results, we assessed nodal metastasis according to clinical tumor size and LVI status (cT ≤ 3 cm with LVI-negative vs. others). pN2-3 was found in 7 (9.3%) of 75 patients with cT ≤ 3 cm with LVI-negative and in 25 (34.7%) of 72 remaining patients (p < 0.001; **Figure 2A**). When analyzed according to age, pN2-3 was observed less frequently in cT ≤ 3 cm and LVI-negative group than in the remaining group; 10.7% vs. 40.0% (p = 0.033) in patients with aged < 50, 8.5% vs. 31.0% (p < 0.001) in patients with aged ≥ 50 (**Figures 2B, C**).

### Axillary pCR in patients who received neoadjuvant chemotherapy

Data were available for 135 patients who received neoadjuvant chemotherapy, followed by surgery, of whom 101 (74.8%) had residual ALN metastasis, and 34 (25.2%) achieved axillary pCR (**Table 3**). All patients with residual ALN metastases underwent ALND. Among the 34 patients with axillary pCR, 21 (61.8%) underwent SLNB, and 13 (38.2%) underwent ALND after SLNB. There were no differences in clinicopathological factors between the two groups, except for TILs.

**Table 3:** Clinicopathologic characteristics of the patients who received neoadjuvant chemotherapy according to axillary lymph node response.

Variables	No axillary pCR (N = 101)	Axillary pCR (N = 34)	Total (N = 135)	P-value
Age				0.270
<50	67 (66.3)	26 (76.5)	93 (68.9)	
≥50	34 (33.7)	8 (23.5)	42 (31.1)	
Breast surgery				0.169
BCS	37 (36.6)	17 (50.0)	54 (40.0)	
Mastectomy	64 (63.4)	17 (50.0)	81 (60.0)	
Axillary surgery				<0.001
SLNB	0	21 (61.8)	21 (15.6)	
ALND	101 (100.0)	13 (38.2)	114 (84.4)	
ER				>0.999^*^
positive	99 (98.0)	34 (100.0)	133 (98.5)	
negative	2 (2.0)	0	2 (1.5)	
PR				0.341
positive	74 (73.3)	22 (64.7)	96 (71.1)	
negative	27 (26.7)	12 (35.3)	39 (28.9)	
TILs^†^				0.001
<20%	50 (75.8)	9 (39.1)	59 (66.3)	
≥20%	16 (24.2)	14 (60.9)	30 (33.7)	
Clinical tumor size				0.103
≤3cm	36 (35.6)	7 (20.6)	43 (31.9)	
>3cm	65 (64.4)	27 (79.4)	92 (68.1)	
Breast pCR				0.497^*^
Yes	8 (7.9)	4 (11.8)	12 (8.9)	
No	93 (92.1)	30 (88.2)	123 (91.1)	

Unless otherwise noted, values are the number of patients, with percentages in parentheses.

^*^P-value was obtained with the Fisher’s exact test.

^†^Missing values.

pCR, pathologic complete response; BCS, breast-conserving surgery; SLNB, sentinel lymph node biopsy; ALND, axillary lymph node dissection; ER, estrogen receptor; PR, progesterone receptor; TILs, tumor-infiltrating lymphocytes.

**Figure 3:**
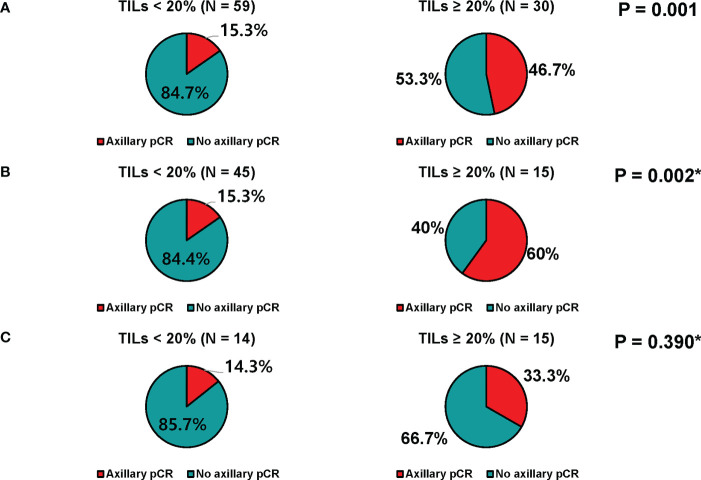
Axillary response of patients who received neoadjuvant chemotherapy. **(A)** All patients, **(B)** patients younger than 50 years, and **(C)** patients of 50 years or older.

TILs could be evaluated in core biopsy samples before neoadjuvant chemotherapy in 89 patients; 59 (66.3%) had low TILs (<20%), and 30 (33.7%) had high TILs (≥20%). Axillary pCR rate was significantly higher in patients with high TILs than those with low TILs (46.7% vs. 15.3%, p=0.001, **Figure 3A**). When stratified by age, similar trends were observed as follows: 60.0% vs. 15.3% (p = 0.002) in patients with aged < 50, 33.3% vs. 14.3% (p = 0.390) in patients with aged ≥ 50 (**Figures 3B, C**). However, the breast pCR rates did not differ according to TILs (**Supplementary Table 1**).

## Discussion

In HR+HER2- breast cancer, multigene assays, such as the Oncotype DX Recurrence Score and MammaPrint, are used to identify patients who do not benefit from chemotherapy, even in those with pN1. Unlike HER2+ breast cancer or TNBC, neoadjuvant chemotherapy is not clearly recommended for patients with HR+HER2- breast cancer who have 1–2 suspicious ALN metastases on pre-treatment breast MRI. In addition, the criteria for chemotherapy based on multigene assays are slightly different depending on age. Therefore, we explored the clinicopathological factors related to the high nodal stage in these populations because chemotherapy should be administered to patients with pN2-3 stratified by age. LVI-positive and large tumor size were associated with a high nodal stage of pN2-3. Although the proportion of pN2-3 was 9.3% among patients with cT ≤ 3 cm and LVI-negative status, it was 34.7% among patients without these factors. Similar results were observed, regardless of age. Our results showed that upfront surgery may be preferable for patients with a cT ≤ 3 cm and LVI-negative status.

In the present study, LVI was the only significant independent factor for multiple ALN metastasis on multivariable analysis. There is concern that it is difficult to accurately diagnose LVI in core biopsy materials owing to retraction artifacts (17). Our study has a fatal drawback in that LVI was analyzed using a surgical specimen rather than a core needle biopsy sample. Previous studies revealed that the concordance rate of LVI was approximately 70% when analyzed using paired samples of core needle biopsy and surgical specimens (18–20). Considering LVI is known to be closely associated with multiple ALN metastases, even in cN0 patients (21, 22), the presence of LVI in a core needle biopsy sample is an important feature that should be included in the standard report to determine the optimal treatment in patients with HR+HER2- breast cancer who have 1–2 suspicious ALNs.

Several previous studies addressed whether the Oncotype DX recurrence score evaluated from biopsy samples was predictive of response to neoadjuvant chemotherapy, but the results were inconsistent (23–27). Moreover, the majority of patients were cN0, and the investigators did not separately evaluate the axillary response in these studies. Furthermore, there is still a concern about the risk of residual axillary lymph node disease mandating ALND after neoadjuvant chemotherapy, even in patients who will elect to undergo chemotherapy due to a high Oncotype DX recurrence score. More data are needed regarding the predictive value of multigene assays on nodal response to neoadjuvant chemotherapy.

The recently reported RxPONDER trial confirmed the chemotherapy benefit in HR+HER2- women under 50 years of age regardless of Oncotype DX recurrence score (13). About half of the women under 50 years of age who had cT ≤ 3 cm and were LVI-negative were node-negative, whereas approximately 75% of the other patients were pN1 or higher. We also confirmed that high TILs (≥20%) were significantly related to axillary pCR in patients who received neoadjuvant chemotherapy, particularly in those with age of < 50. Accordingly, neoadjuvant chemotherapy is more likely beneficial for patients under 50 years of age who have cT > 3 cm or are LVI-positive, especially with high TILs.

In line with previous studies (3, 4), breast pCR was low (approximately 9%) among patients who received neoadjuvant chemotherapy regardless of TILs. Although it is generally known that the axillary response is better than the breast response (28, 29), there are limited data on the difference between breast and axillary pCR according to TILs in HR+HER2- breast cancer. Our study showed that axillary pCR was relatively high at 47% among patients with high TILs, which accounted for approximately 35% of the population. This is an encouraging result that neoadjuvant chemotherapy provides the option to omit ALND when TILs levels are high, even in HR+HER2- breast cancer. The higher axillary response in patients with high TILs might be due to several reasons. First, as immune system activation would be significantly more effective in the lymph nodes than in the breast tissue, neoadjuvant chemotherapy could enhance the axillary response versus the breast response (30). Second, this outcome may occur because there are more luminal B subtypes in patients with high TILs since axillary pCR is higher in luminal B than in luminal A breast cancer (29).

The major limitation of our study was its retrospective design and relatively small cohort derived from a single institution. This is an unavoidable aspect that arises from including patients with HR+HER2- breast cancer who have only 1–2 suspicious ALNs, which is uncommonly encountered in clinical practice. Another limitation is that we evaluated LVI in surgical specimens and TILs in core needle biopsy samples. Finally, the majority of patients did not receive the FNAB. Nevertheless, it is assumed that FNAB may be helpful to preclude the multiple ALN metastases group because the multiple ALN metastases rate was relatively low in patients with FNAB-negative who received upfront surgery. Further studies are required to confirm the reproducibility of our findings in a large cohort in which all pathological factors are examined in core needle biopsy or FNAB samples.

In summary, the pN2-3 rate was low in patients with cT ≤ 3 cm and LVI-negative status. Given that chemotherapy can be avoided based on multigene assays, upfront surgery is an appropriate treatment option, especially for women aged > 50 years. In contrast, among the patients with high TILs who had cT > 3 cm or LVI-positive status, neoadjuvant chemotherapy rather than upfront surgery should be considered because the omission of ALND is expected owing to a relatively high axillary pCR. Our findings may help determine the optimal treatment strategy for patients with HR+HER2- breast cancer and 1–2 suspicious ALNs on pre-treatment breast MRI.

## Data availability statement

The original contributions presented in the study are included in the article/**Supplementary Material**. Further inquiries can be directed to the corresponding author.

## Ethics statement

The studies involving human participants were reviewed and approved by the study protocol was reviewed and approved by the Institutional Review Board (IRB) of Gangnam Severance Hospital, Yonsei University, Seoul, Korea and adhered to the tenets of the Declaration of Helsinki (IRB no. 3-2021-0155). The IRB waived the requirement for written informed consent owing to the study’s retrospective design. Written informed consent for participation was not required for this study in accordance with the national legislation and the institutional requirements.

## Author contributions

Study conception and design: SL, SB. Acquisition of data: SL, SA, JJi, YK, JJa, SB. Drafting of the article: SL, JJe, SB. Data analysis and interpretation: SL, SA, JJe, SB. All authors contributed to the article and approved the submitted version.
