# Supplementary material for: Optimal treatment strategy for hormone receptor-positive human epidermal growth factor receptor 2-negative breast cancer patients with 1–2 suspicious axillary lymph node metastases on breast magnetic resonance imaging: upfront surgery vs. neoadjuvant chemotherapy
Source: Front Oncol. 2023 May 17;13:936148. doi: 10.3389/fonc.2023.936148 (PMC10230027; doi:10.3389/fonc.2023.936148)
Supplement: Supplementary file 1 [file Table_1.docx]

**Supplementary Table 1. The results of fine-needle aspiration biopsy**

| Upfront surgery | Not done  (n = 102) | Negative  (n = 17) | Positive  (n = 24) | Inconclusve^*^  (n = 4) | P-value |
| --- | --- | --- | --- | --- | --- |
| pN0 | 43 (42.2) | 9 (52.9) | 0 | 3 (75.0) | <0.001^†^ |
| pN1 | 39 (38.2) | 7 (41.2) | 13 (54.2) | 1 (25.0) |  |
| pN2-3 | 20 (19.6) | 1 (5.9) | 11 (45.8) | 0 |  |
| Neoadjuvant chemotherapy | Not done  (n = 84) | Negative  (n = 5) | Positive  (n = 43) | Inconclusve^*^  (n = 3) | P-value^†^ |
| Axillary pCR | 60 (71.4) | 2 (40.0) | 37 (86.0) | 2 (66.7) | 0.059 |
| Axillary non-pCR | 24 (28.6) | 3 (60.0) | 6 (14.0) | 1 (33.3) |  |

Unless otherwise noted, values are the number of patients, with percentages in parentheses.

^*^Pathologic evaluation was failed due to cell paucity.

^†^P-value was obtained with the Fisher’s exact test.

**Supplementary Table 2. Breast pCR according to TILs stratified by age.**

|  |  | TILs < 20% | TILs ≥ 20% | P-value^*^ |
| --- | --- | --- | --- | --- |
| All | Breast pCR |  |  | 0.113* |
|  | Yes | 3 (5.1) | 5 (16.7) |  |
|  | No | 56 (94.9) | 25 (83.3) |  |
| Age < 50 | Breast pCR |  |  | 0.732 |
|  | Yes | 2 (.4) | 1 (6.7) |  |
|  | No | 43 (95.6) | 14 (93.3) |  |
| Age ≥ 50 | Breast pCR |  |  | 0.330 |
|  | Yes | 1 (7.1) | 4 (26.7) |  |
|  | No | 13 (92.9) | 11 (73.3) |  |

^*^*P*-value was obtained with the Fisher’s exact test.

pCR = pathologic complete response, TILs = tumor-infiltrating lymphocytes
